# Characterization and reduction of non-endocrine cells accompanying islet-like endocrine cells differentiated from human iPSC

**DOI:** 10.1038/s41598-022-08753-5

**Published:** 2022-03-18

**Authors:** Hideyuki Hiyoshi, Kensuke Sakuma, Noriko Tsubooka-Yamazoe, Shinya Asano, Taisuke Mochida, Junji Yamaura, Shuhei Konagaya, Ryo Fujii, Hirokazu Matsumoto, Ryo Ito, Taro Toyoda

**Affiliations:** 1 T-CiRA Discovery, Research, Takeda Pharmaceutical Company Limited, 26-1, Muraoka-Higashi 2-chome, Fujisawa, Kanagawa 251-8555 Japan; 2 Department of Cell Growth and Differentiation, Center for iPS Cell Research and Application (CiRA), Kyoto University, 53 Kawahara-cho, Shogoin, Sakyo-ku, Kyoto, 606-8507 Japan; 3 Takeda-CiRA Joint Program for iPS Cell Applications (T-CiRA), Fujisawa, Kanagawa Japan; 4 Orizuru Therapeutics, Inc, Fujisawa, Kanagawa Japan; 5 Axcelead Drug Discovery Partners, Inc, Fujisawa, Kanagawa Japan; 6 Pharmaceutical Science, Takeda Pharmaceutical Company Limited, Fujisawa, Kanagawa Japan

**Keywords:** Regenerative medicine, Stem-cell biotechnology, Tissue engineering, Metabolic disorders

## Abstract

The differentiation of pancreatic endocrine cells from human pluripotent stem cells has been thoroughly investigated for their application in cell therapy against diabetes. Although non-endocrine cells are inevitable contaminating by-products of the differentiation process, a comprehensive profile of such cells is lacking. Therefore, we characterized non-endocrine cells in iPSC-derived pancreatic islet cells (iPIC) using single-cell transcriptomic analysis. We found that non-endocrine cells consist of (1) heterogeneous proliferating cells, and (2) cells with not only pancreatic traits but also liver or intestinal traits marked by *FGB* or *AGR2*. Non-endocrine cells specifically expressed *FGFR2*, *PLK1*, and *LDHB*. We demonstrated that inhibition of pathways involving these genes selectively reduced the number of non-endocrine cells in the differentiation process. These findings provide useful insights into cell purification approaches and contribute to the improvement of the mass production of endocrine cells for stem cell-derived cell therapy for diabetes.

## Introduction

Type 1 diabetes mellitus is an autoimmune disorder in which insulin-secreting pancreatic β-cells are destroyed, leading to uncontrolled hyperglycemia and hypoglycemic event, and devastating multiple complications^1^. To cure patients with this disease, whole-organ pancreas transplantation and pancreatic islet transplantation from deceased donors are effective therapeutic options^2,3^. However, due to donor shortages and gradual loss of graft function, the development of alternative and unlimited cell sources is urgently needed. As such an alternative, the development of pancreatic islet-like endocrine cells differentiated from pluripotent stem cells (PSC), such as induced pluripotent stem cells (iPSC) and embryonic stem cells (ESC), is anticipated^4,5^.

Multiple studies have demonstrated the in vitro generation of pancreatic endocrine cells from human iPSC and ESC by stepwise differentiation recapitulating the developmental process^6–10^. Implantation of the generated cells normalizes hyperglycemia associated with insulin secretion from grafts in rodent type 1 diabetes models. However, the induction efficiency at each differentiation stage was not 100%, and non-endocrine cells are inevitably included in the generated cells as by-products, potentially causing unwanted outcomes. In fact, implanted cells containing 2–10% non-endocrine cells^11,12^ proliferated to form cysts and enlarged grafts by > tenfold within 200 days after implantation in 8/8 animals^13^. Therefore, approaches to reduce or exclude contaminating non-endocrine cells are desirable for clinical applications.

Given that > 1 × 10^8^ cells are used for current islet transplantation protocols^14^, a similar number of cells is assumed to be required for iPSC/ESC-derived pancreatic islet-like cells. Feasible candidate approaches to reduce the number of unintended cells from over hundred million in vitro-generated cells include compound treatments or metabolic selection based on properties specific to the relevant cells. One recently reported suitable candidate is verteporfin, a Yes-associated protein (YAP) inhibitor, which indirectly reduces the number of pancreatic progenitor cells by promoting the induction of pancreatic endocrine cells^15^. However, non-endocrine cells are likely to include not only pancreatic progenitors but also pancreatic exocrine cells, pancreatic stellate cells, and potentially even cells of other lineages. In contrast to endocrine cells, non-endocrine cells have not been fully characterized. Therefore, the purpose of this study was to characterize non-endocrine cells at single-cell resolution and to apply the information obtained to develop novel methods for the reduction or exclusion of non-endocrine cells.

## Results

### Subcutaneously engrafted iPIC showed long-term efficacy and morphology maintenance

**Figure 1:**
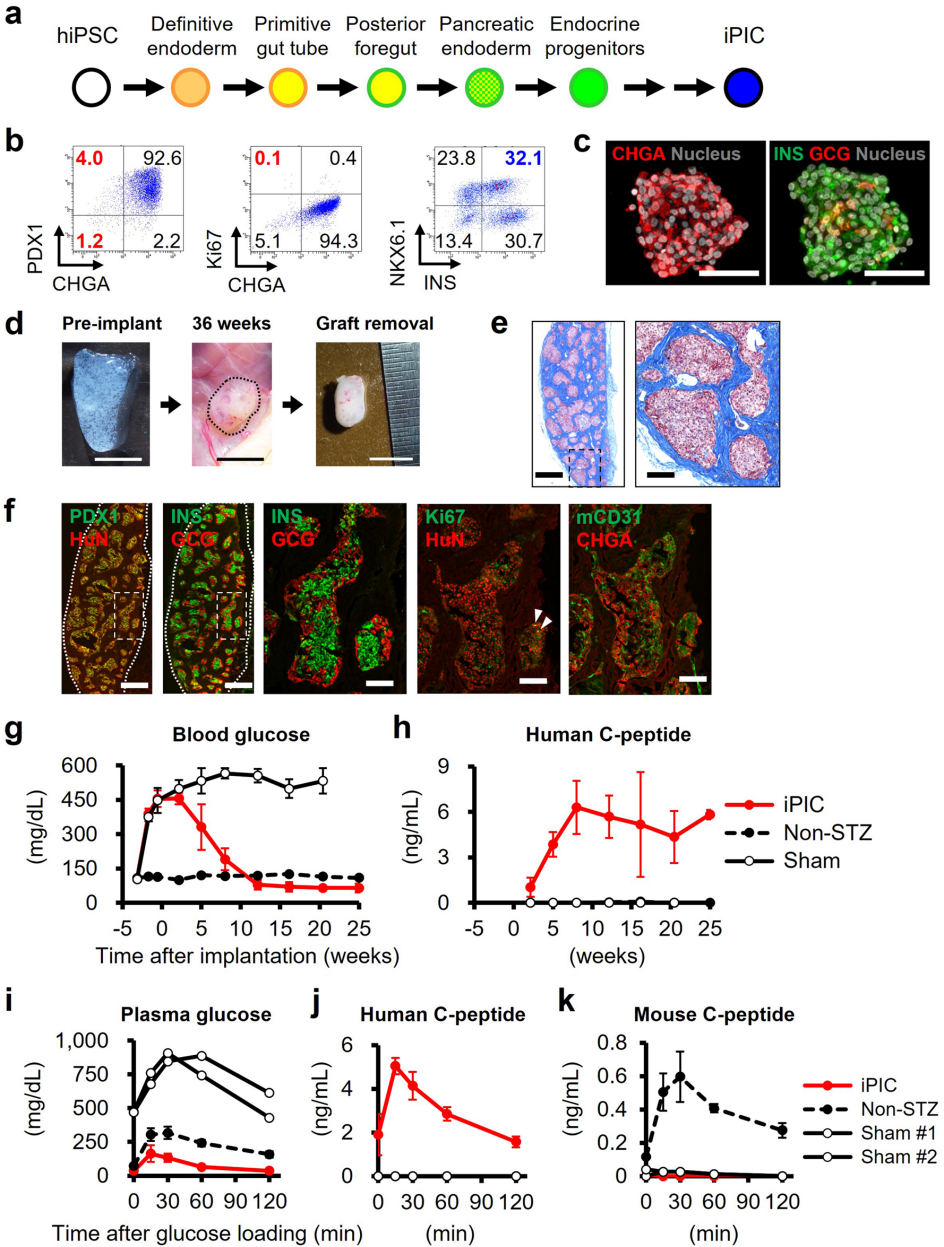
Generation and subcutaneous implantation of iPIC. (**a**) Schematic representation of iPIC differentiation from human iPSC. (**b**) Representative flow cytometry plots illustrating the protein expression of iPIC. The presented plots are those of samples for single-cell RNA sequencing. (**c**) Representative immunohistochemistry images of iPIC aggregates before implantation. White bars indicate 50 μm. (**d**–**k**) Cell implantation experiments in STZ-NOD-scid mice. Mice were implanted with iPIC (3–4 × 10^6^ cells/mouse) embedded in fibrin gel into the subcutaneous space (iPIC). Sham-operated STZ-NOD-scid mice (Sham) and non-STZ control mice (Non-STZ) were also prepared. (**d**) Macroscopic photographs of iPIC embedded in fibrin gel before implantation and the iPIC grafts subcutaneously engrafted or retrieved 36 weeks after implantation. White and black bars indicate 5 mm. Images are representative of six samples showing similar results. (**e**) Masson's trichrome-stained section 25 weeks after implantation. The collagen-rich fibrotic regions are stained blue. Black bars indicate 500 μm at low magnification and 100 μm at high magnification. Images are representative of six samples showing similar results. (**f**) Graft immunohistochemistry images 25 weeks after implantation. White bars indicate 500 μm at low magnification in the left two images and 100 μm at high magnification in the right three images. White arrowheads indicate Ki67^+^HuN^+^ cells. HuN; human nucleus. Images are all taken from the same sample and are representative of six samples showing similar results. (**g**,**h**) Blood glucose (**g**) and plasma human C-peptide (**h**) levels after iPIC (3 × 10^6^ cells/mouse) implantation. Data are shown as the mean ± SD (iPIC; n = 5 → 3, Non-STZ; n = 4 → 3, sham; n = 4 → 3). The decrease in n number is due to unexpected death. (**i**–**k**) Plasma glucose (**i**), human C-peptide (**j**) and mouse C-peptide (**k**) levels during the oral glucose tolerance test at 23 weeks after implantation. Data are shown as the mean ± SD (iPIC; n = 4, Non-STZ; n = 3, sham; individual data in n = 2).

With reference to landmark reports describing the differentiation protocol for human PSC-derived endocrine cells^6,7,9,10^, we generated iPSC-derived pancreatic islet cells (iPIC) from the Ff-I14s04 and QHJI-14s04 iPSC lines using a 7-step in vitro differentiation protocol (Fig. 1a and Supplementary Fig. 1). In iPIC, ~ 95% of cells were positive for the endocrine cell marker CHGA, more than 95% of cells were positive for the pancreatic cell marker PDX1, and few CHGA^-^ cells expressed the proliferative marker Ki67 (0.1%) (Fig. 1a,c). Approximately 30–35% of cells were either NKX6.1^+^INS^+^ or NKX6.1^−^INS^+^, and these cells were expected to mature into β and α cells, respectively, after implantation^7,16,17^ (Fig. 1b,c).

To investigate the cell maturation and function of iPIC in vivo, we implanted iPIC embedded in fibrin gel, which is known to support the engraftment of porcine islets^18,19^, into the subcutaneous spaces of mice with streptozotocin-induced diabetes. The grafts of iPIC formed granulation tissue and could be easily retrieved from the host 25–36 weeks after implantation (Fig. 1d). The graft size was less than 5 mm in diameter, which was not larger than the mixture of iPIC and fibrin gel before implantation, in all mice. Histological analysis showed that the grafts were composed of compact endocrine cell clusters (50–500 μm) surrounded by host-derived fibrous tissues (Fig. 1e,f). The endocrine cell clusters were characterized by the arrangements of INS^+^ cells in the core and GCG^+^ cells in the periphery, similar to the structure of prenatal fetal human islets and adult rodent islets^20,21^. While the proliferation of non-target cells was not observed throughout the graft, endocrine clusters contained a few Ki67^+^HuN^+^ proliferative cells (Fig. 1f). This is within the normal range of cell turnover, given that a few Ki67^+^ cells have been reported in endogenous islets in vivo^22,23^. Infiltration of host-derived blood vessels into the endocrine clusters was evidenced by the presence of mouse CD31^+^ cells and red blood cells (Fig. 1e,f). Consistent with histological observations, eight weeks after implantation, human C-peptide secretion from implanted iPIC plateaued at a high level (6.3 ± 1.8 ng/mL), followed by return to a normoglycemic state (Fig. 1g,h). To evaluate insulin secretion in response to glucose levels, we performed an oral glucose tolerance test 23 weeks after implantation and found that plasma human C-peptide levels were increased within 15 min after glucose loading (Fig. 1i,j). This rapid response was comparable or superior to that of endogenous insulin secretion from mouse islets (Fig. 1k). The blood glucose levels in iPIC implanted mice were lower than those in non-STZ control mice (Fig. 1g), which is possibly attributed to the lower blood glucose setpoint by human islets than by mouse islets in rodent^13,24^. Nevertheless, iPIC-implanted mice showed body weight transition equivalent to that of non-STZ control mice (Supplementary Fig. 2) with no abnormalities in physical conditions. These results suggested that iPIC maintained the function of blood glucose normalization for months in vivo without morphological abnormality.

### Classification of cells composing iPIC to expose potential remaining non-endocrine cells

**Figure 2:**
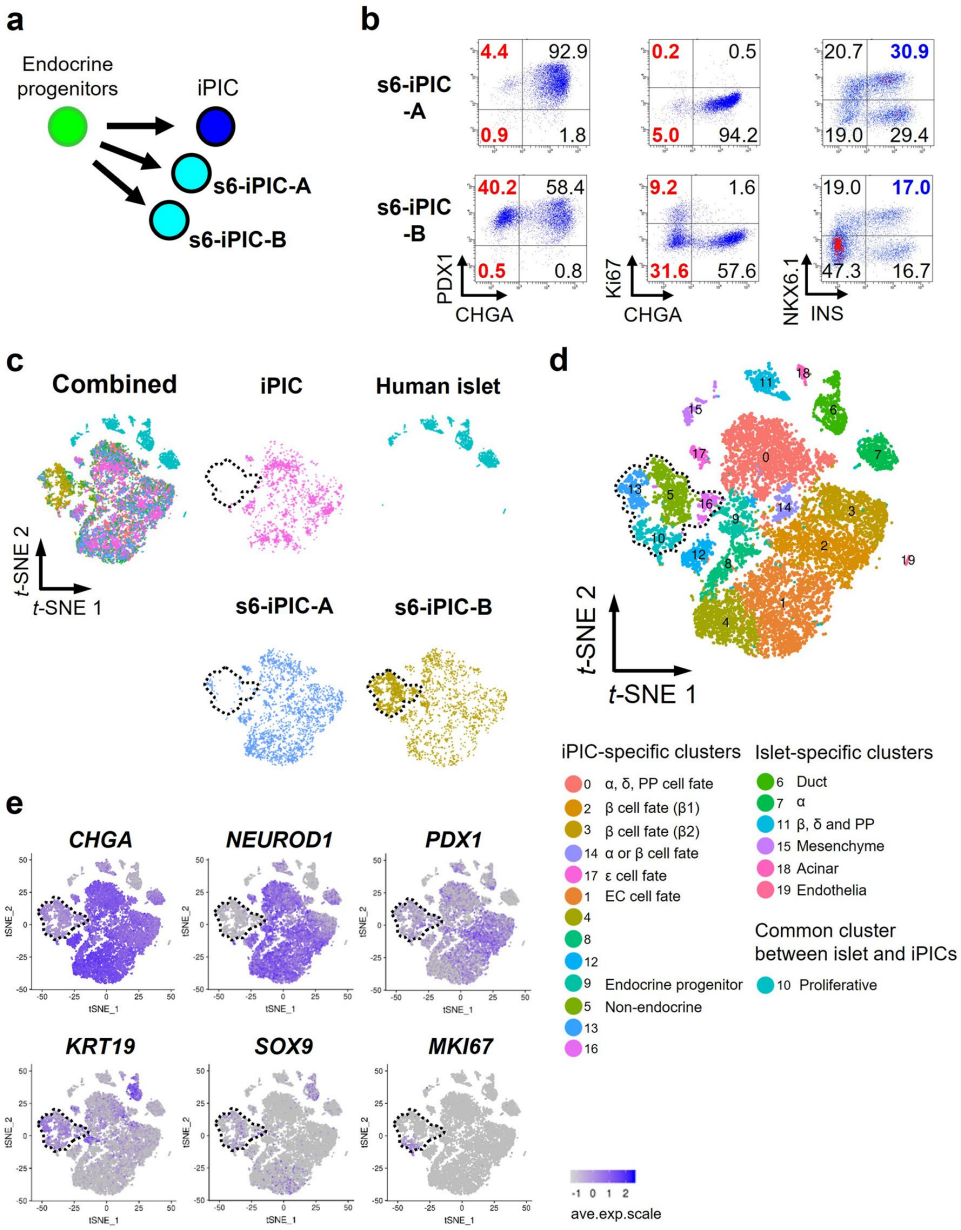
Classification of the cells composing iPIC and extraction of non-endocrine cells through single-cell RNA sequencing. (**a**) Schematic representation of three versions of iPICs differentiation subjected to single-cell RNA sequencing. (**b**) Representative flow-cytometry plots illustrating the protein expression of s6-iPIC-A (upper) and s6-iPIC-B (lower). The presented plots are those of samples for single-cell RNA sequencing. (**c**) A combined cell distribution from a total of six samples and individual cell distributions. The six samples are as follows: 1 sample of iPIC, 3 samples of s6-iPIC-A, 1 sample of s6-iPIC-B, and 1 sample of reference human islet. For s6-iPIC-A, only a representative sample of the 3 samples is shown. For the remaining two samples of s6-iPIC-A, individual *t-*SNE projects are shown in Supplementary Fig. 3a. The dotted line in black represents the non-endocrine (*CHGA*^*-*^*NEUROD1*^*-*^) population from the iPICs, as shown in (**e**). (**d**) Shared nearest neighbors clustering identified fourteen cell clusters for in vitro differentiated iPICs (0–5, 8–10, 12–14, 16, and 17) and seven clusters for human islet samples (6, 7, 10, 11, 15, 18, and 19). Clusters 1, 4, 8, and 12 are all EC cell fate, while Clusters 5, 13, 16 are all non-endocrine cell. The dotted line in black represents the non-endocrine population (Clusters 5, 13, 16, and *CHGA*^-^ population of Cluster 10) from the iPICs. (**e**) Single-cell gene expression of representative markers related to the pancreas, endocrine cells and proliferation in combined *t*-SNE projections. The dotted line in black represents the non-endocrine population from the iPICs.

Based on the results of the in vivo experiments, the risk of contaminating non-endocrine cells was relatively low in iPIC. However, considering the cell number (> 1 × 10^8^) assumed to be needed for implantation in patients^14^, it is reasonable to pursue an understanding of, and methods for reducing, potentially contaminating cells as much as possible, to minimize future clinical risk levels. To expose potentially contaminating non-endocrine cells, we profiled the single-cell transcriptomes of iPIC. However, since the percentage of non-endocrine cells in iPIC was approximately 5% (Fig. 1b), we assumed that some contaminating cells could fail to be detected by single-cell RNA sequencing (scRNA-seq). Therefore, to widen the variety of potentially contaminating cells, we analyzed not only iPIC but also two types of reference cell: iPIC that were not treated with stage 7 differentiation factors (s6-iPIC-A), and s6-iPIC-A derivative with omission of a differentiation factor PD-166866 (s6-iPIC-B) (Fig. 2a and Supplementary Fig. 1). The proportions of CHGA^-^ and CHGA^-^Ki67^+^ cells were comparable in s6-iPIC-A (5.2% and 0.2%) and increased in s6-iPIC-B (40.8% and 9.2%) compared to iPIC (Figs. 1b and 2b).

We collected sequence data from 19,969 cells, including the three versions of iPICs and the reference human islet sample. After quality control, we performed unsupervised cell clustering and visualization on the data from the remaining 17,100 cells (Fig. 2c,d). The cells in the different induction batches of s6-iPIC-A were all classified into the same cell clusters with a slight percentage variation, indicating that there was no prominent batch effect in the differentiation protocol (Supplementary Fig. 3).

Next, we classified the cell clusters based on pancreas-related gene expression (Fig. 2d and Supplementary Fig. 4). In iPICs, we observed three major CHGA^high^ endocrine cell identities as previously reported^11,25,26^: (i) β-cell fate cells (Clusters 2 and 3); (ii) a mixture of α-, δ-, PP- and G-cell fate cells^27^ (Cluster 0); and (iii) enterochromaffin (EC)-cell fate cells (Clusters 1, 4, 8 and 12). There were three other endocrine clusters: ε-cell fate cells (Cluster 17), endocrine progenitors that express *NGN3* (Cluster 9), and intermediate cells for β- or α- cells (Cluster 14)*.* Conversely, Clusters 5, 13, and 16, and most of Cluster 10 were assigned to non-endocrine cells with low expression of the endocrine markers *CHGA* and *NEUROD1* (inside the black dotted line in Fig. 2d,e). These non-endocrine cell populations expressed *PDX1*, *KRT19*, and *SOX9*, as did pancreatic duct cells (Cluster 6) in human islets (Fig. 2e), while *PRSS1* and *CPA1*, which are strongly expressed in acinar cells (Cluster 18), were not expressed (Supplementary Fig. 4a). Considering that most CHGA^-^ cells were PDX1^+^ in flow cytometry analysis of iPICs (Fig. 2b), non-endocrine cells were likely to be cells of the developing pancreatic duct or surrounding tissues. In terms of the residual degree of these non-endocrine cell populations, we could not detect any difference between s6-iPIC-A and iPIC in flow cytometry analysis (Fig. 2b), but found that in scRNA-seq, the residual cells of Cluster 5 and 13 were higher in s6-iPIC-A than in iPIC (Fig. 2c and Supplementary Fig. 3b). Thus, using iPICs with different degrees of non-endocrine cells, we could extract non-endocrine cells at single-cell resolution, and found that these cells were reduced gradually in the process leading to current best-practice iPIC.

### Characterization of non-endocrine cell populations and detection of non-endocrine subpopulations with quantitative PCR

To evaluate non-endocrine cell populations in multilateral ways, we analyzed scRNA-seq data using several methods. First, to define the developmental hierarchy of non-endocrine cells, reconstruction of cells consisting of iPICs was performed by trajectory analysis using Monocle 2^28–30^. Trajectory projections of all types of iPICs showed only one branching point (Supplementary Fig. 5a). Given that endocrine cells are mature cell types, Clusters 5, 10, 13, and 16 were projected to be more immature progenitor-like populations. Next, to evaluate proliferative activity, we performed cell cycle phase assignments based on S-phase, G2 and M gene signatures, and observed high S-scores in Clusters 5, 10, 13, and 16 (Supplementary Fig. 5b). In particular, Cluster 10 had a high G2M-score and expressed *MKI67* (Fig. 2e). Of note, the lower tip of Cluster 10 in Fig. 2d was actually *CHGA*^+^ and *NEUROD1*^+^ proliferative cells (Fig. 2e). Thus, Cluster 10 was a heterogeneous population of proliferating cells, including a few replicating endocrine cells in iPICs and human islets (0.8%, 19 out of 2,419 cells) (Supplementary Fig. 3b). This proportion of proliferating cells in endogenous human islet is consistent with previous reports^22,23^.

**Figure 3:**
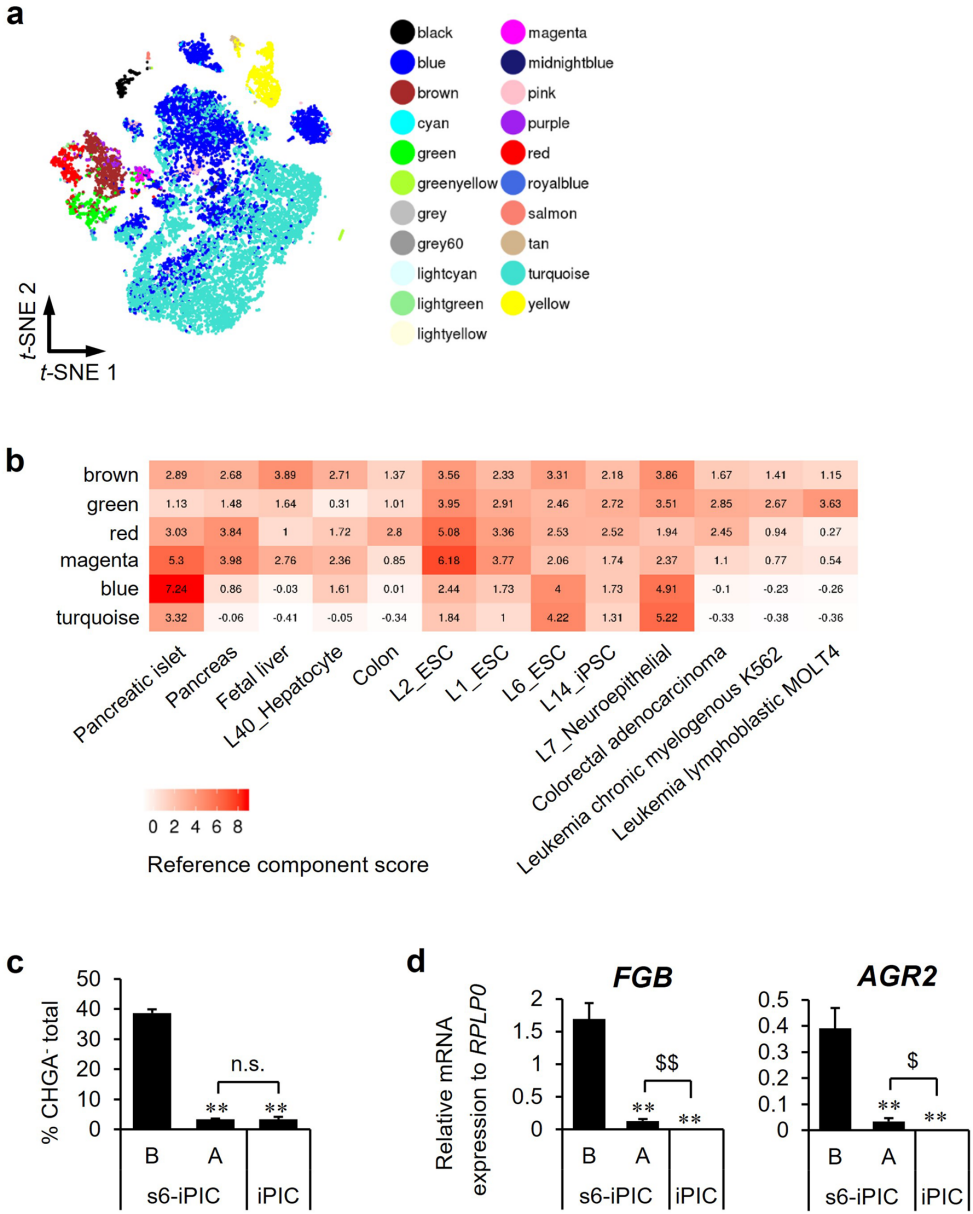
Detailed analysis focusing on non-endocrine cells and validation of identified markers for detecting non-endocrine subpopulations. (**a**) Newly classified cell populations by reference component analysis (RCA) on the *t-*SNE projection. (**b**) A heatmap of tissues and cell lines with high similarity (reference component score > 2.5) to non-endocrine cells (brown, green, red and magenta). See Supplementary Fig. 6b for the full RCA heatmap. (**c**) The proportion of putative non-endocrine cells (CHGA^-^) for differentiated s6-iPIC-B, s6-iPIC-A and iPIC by flow cytometry analysis. Data are shown as the mean ± SD (n = 4, technical replicates). Reproducibility confirmed by 3 independent experiments. **P < 0.01 versus s6-iPIC-B, Dunnett's test. n.s.; not significant, Aspin-Welch *t-*test. (**d**) Validation of the identified novel non-endocrine markers in quantitative real-time PCR experiments. Expression levels were normalized to *RPLP0* and are shown as the mean ± SD (n = 4, technical replicates). Reproducibility confirmed by 3 independent experiments. **P < 0.01 versus s6-iPIC-B, Dunnett's test. ^$$^*P* < 0.01, ^$^*P* < 0.05, Student t-test.

Subsequently, to clarify the characteristics of each non-endocrine cell cluster, we performed reference component analysis (RCA), which indicates transcriptome similarity to known tissues or cell lines^31^. Non-endocrine cell populations (Clusters 5, 13, 16, and *CHGA*^-^ population of Cluster 10) were again distinct from endocrine cells in RCA (Fig. 3a and Supplementary Fig. 6a). Among non-endocrine cell populations, Cluster 16 indicated in magenta scored higher for pancreas and pancreatic islets than Clusters 5, 13, and *CHGA*^-^ population of Cluster 10 (brown, red, and green) (Fig. 3b and Supplementary Fig. 6b). In addition, the expression intensity of *YAP1*, which is known to be repressed before endocrine specification^32^, was lower in Cluster 16 than in the other non-endocrine clusters (Supplementary Fig. 4a). Therefore, Cluster 16 might be the transitional state leading to the neighboring Cluster 9, an *NGN3*^+^ endocrine progenitor population. The high score for tumor cells in *CHGA*^-^ population of Cluster 10 (green) is consistent with the idea that Cluster 10 was a heterogeneous population of proliferating cells (Fig. 3b). Notably, high scores for ESC and the neuroepithelia were also indicated not only in the non-endocrine clusters but also in the endocrine populations in human islets and iPICs (blue and turquoise). Clusters 5 (brown) and 13 (red) scored high for the fetal liver and colon, respectively, in addition to the pancreas (Fig. 3b and Supplementary Fig. 6c). In line with these results, Cluster 5 contained liver-related genes such as *FGB*, *GSTA1*, *AFP*, *APOC1* and *APOA*, and Cluster 13 contained intestine-associated *AGR*^33^ among the top 10 differentially expressed genes (DEGs) (Supplementary Fig. 4b). These results suggest that cells in Clusters 5 and 13 are basically pancreatic linage, but partially have liver or intestinal traits.

Although scRNA-seq revealed that the proportion of non-endocrine cells in Clusters 5 and 13 was reduced in iPIC compared to s6-iPIC-A (Fig. 2c and Supplementary Fig. 3b), flow cytometry analysis did not distinguish s6-iPIC-A and iPIC in terms of the remaining non-endocrine cells (Fig. 3c). To detect the proportion of non-endocrine cells by means other than scRNA-seq that takes several weeks to assess cells, we performed quantitative PCR (qPCR) for *FGB* and *AGR2*, which were specific genes in the highest DEGs for Clusters 5 and 13, respectively (Supplementary Fig. 4b). The expression of *FGB* and *AGR2* was significantly lower in iPIC than in s6-iPIC-A (Fig. 3d). Of note, the expression of *FGB* and *AGR2* was not detected in two out of three independent qPCR measurements in iPIC using 1–3 × 10^4^ cells, demonstrating that Cluster 5 or 13 cells hardly remained in iPIC. These results indicate that qPCR for *FGB* and *AGR2* is practical and sensitive for reliably detecting residual non-endocrine cell presence instead of scRNA-seq.

### Novel approaches to reducing the number of proliferative non-endocrine cells

**Figure 4:**
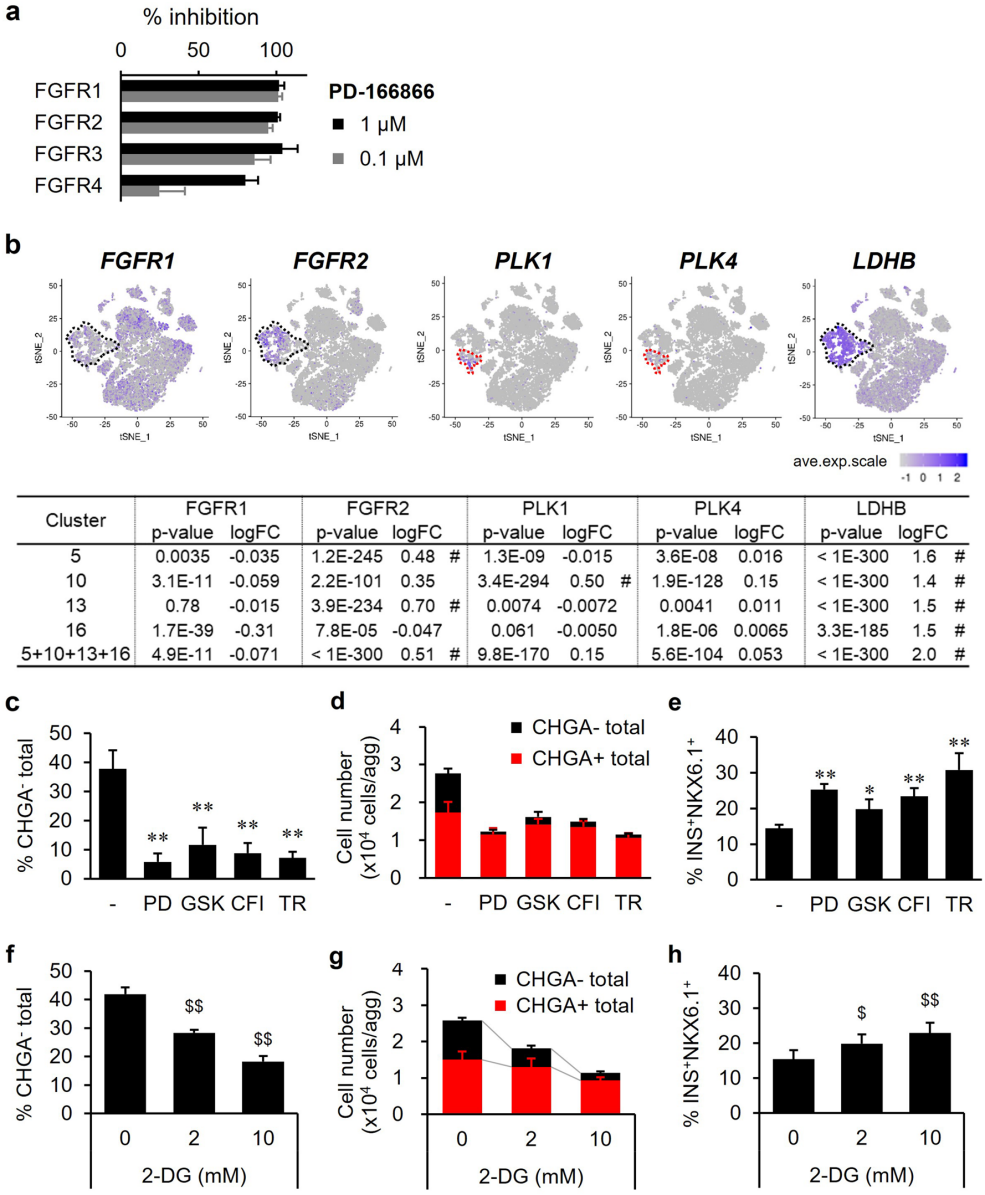
Reduction of the number of non-endocrine cells by inhibition of non-endocrine specific factors. (**a**) Inhibitory activities of PD-166866, known as a selective FGFR1 inhibitor, for all four FGF receptor isoforms in the TR-FRET-based competitive binding assay. Data are shown as the mean ± SD (n = 4, collected from independent experiments). (**b**) Single-cell gene expression analysis showing that *FGFR1*, *FGFR2*, *PLK1*, *PLK4* and *LDHB* were specifically expressed in non-endocrine clusters. The dotted line in black represents the non-endocrine population from the iPICs. The dotted line in red represents proliferative cluster 10. The table contains the *p*-value and fold change for each indicated non-endocrine cluster compared to the others. # for *p*-values less than 0.05 and fold changes more than 1.5. See Supplementary Fig. 8a for single cell gene expression of other *FGFR* and *PLK* isoforms. (**c–h**) Evaluation of the non-endocrine cell reduction effects of kinases and glycolytic inhibitors. S6-iPIC-B cells were additively treated with the indicated inhibitors from day 4 at stage 6 for purification and then collected and analyzed for the ratios of putative non-endocrine cells (CHGA^-^) (**c,f**), cell number (**d,g**) and the ratios of putative β-cells (INS^+^NKX6.1^+^) (**e,h**). Data are shown as the mean ± SD (n = 4–5, collected from independent experiments). **P < 0.01 versus s6-iPIC-B, Dunnett's test. ^$^*P* < 0.025, ^$ $^ *P* < 0.005, versus s6-iPIC-B, one-tailed Williams’ test. PD; PD-166866, GSK; GSK 461364, CFI; CFI-400945, TR; TR06141363, 2-DG; 2-deoxy-D-Glucose.

Finally, we explored novel approaches to reduce the number of non-endocrine cells. First, we scrutinized the removal effect of PD-166866 on non-endocrine cells. PD-166866, which was used to induce s6-iPIC-A and iPIC, not only reduced non-endocrine cells in vitro (Fig. 2b), but also suppressed graft hypertrophy in vivo while maintaining glycemic control activity (Supplementary Fig. 7a–c). PD-166866 is a well-known selective inhibitor of FGFR1^34,35^. To clarify the targets of PD-166866, we assessed the inhibition profile of PD-166866 for FGFR isoforms using a global kinase panel assay^36^. We found that PD-166866 inhibited all FGFR isoforms in the micromolar range (Fig. 4a), suggesting that PD-166866 is a pan-FGFR inhibitor. We then extracted the expression profile of *FGFR isoforms* in all types of iPICs from the scRNA-seq data. While *FGFR2* was dominantly expressed in non-endocrine cell subpopulations, *FGFR1*, *FGFR3* and *FGFR4* were ubiquitously expressed (Fig. 4b and Supplementary Fig. 8a). In addition, PD-166866 reduced only the number of non-endocrine cells without affecting endocrine cells (Fig. 4c,d). These results suggest that PD-166866 reduced the number of non-endocrine cells mainly via FGFR2 inhibition in non-endocrine cells.

Next, we investigated whether inhibition of the cell cycle reduces the non-endocrine cell populations, because activation of the cell cycle was observed in these populations (Supplementary Fig. 5b). As cell cycle inhibitors, we selected polo-like kinase (PLK) inhibitors, for which clinical development as anticancer agents is progressing^37^. By extracting the expression of *PLK isoforms* in all types of iPIC, we found that *PLK1* and *PLK4* were highly expressed in Cluster 10 (Fig. 4b and Supplementary Fig. 8a). We then evaluated whether the PLK1 inhibitor GSK 461364 and the PLK4 inhibitor CFI-400945 reduced the number of non-endocrine cells. In a similar manner, we tested the multi-kinase inhibitor TR06141363, which inhibits PLK4 and FGFR isoforms (Supplementary Fig. 8b) and was added to the induction step of iPIC generation. All the compounds with PLK-inhibitory activity reduced non-endocrine cells to the same extent as PD-166866 (Fig. 4c). Notably, these compounds selectively reduced non-endocrine cells with little reduction in endocrine cells (Fig. 4d). Accordingly, the proportion of NKX6.1^+^INS^+^ cells increased (Fig. 4e). In addition, using a global kinase panel assay, we validated that the PLK1 inhibitor GSK 461364 had almost no inhibitory activity against FGFR isoforms in the micromolar range (Supplementary Fig. 8b). These results suggest that PLK inhibition selectively reduces the number of non-endocrine cells via a mechanism different from FGFR inhibition.

For the other novel approach, we focused on *LDHB*, which was included in the top 10 significant DEGs of Clusters 5, 10 and 13 (Supplementary Fig. 4b). Based on the high expression of *LDHB* in non-endocrine cells (Fig. 4b), we hypothesized that a dependency on glycolysis as an energy production pathway is more prevalent in non-endocrine cells than in endocrine cells. Therefore, we examined whether 2-deoxy-D-glucose (2-DG), which can broadly inhibit glycolysis^38^, reduces the number of non-endocrine cells. Treatment with 2-DG significantly reduced the number of non-endocrine cells and increased the proportion of NKX6.1^+^INS^+^ cells in a dose-dependent manner (Fig. 4f,g,h). This treatment also decreased the number of endocrine cells, but the reduction rate of non-endocrine cells was higher (80.7 ± 2.6% at 10 mM) than that of endocrine cells (37.4 ± 6.9% at 10 mM) (Fig. 4g). These results support the idea that glycolysis is more active in non-endocrine cells than in endocrine cells, and inhibition of glycolysis is a potential target for the reduction of unintended non-endocrine cells.

## Discussion

Non-endocrine cells account for a small proportion of iPSC-derived islet cells, but their characteristics are significant for cell therapy against type 1 diabetes. In the present study, we analyzed three types of iPICs with different degrees of non-endocrine cell presence and exposed the potential remaining non-endocrine cells in iPIC. Our single-cell dataset categorized non-endocrine cells into 1) heterogeneous proliferating cells, and 2) cells with high levels of liver- or colon-related genes despite co-expression of pancreatic markers (Figs. 2b,e, and 3a). We speculated that the latter cell population was fundamentally pancreatic, but partially retained traits of either liver or intestinal lineages; these are spatiotemporally close to the pancreatic region during development. It is reasonable to attribute the presence of these cells to artificial stepwise differentiation in vitro, as the in vitro environment probably lacks unknown factors that are needed to stabilize the pancreatic developmental process in vivo. Nonetheless, it is worth noting that maturation to liver or intestinal cell types was not apparent in implanted iPIC grafts (Fig. 1e,f), indicating that there was little contamination with cells displaying multiple tissue traits or that the cells are prone to lose cell fates other than pancreas, at least on the scale of this study.

The presence of non-endocrine cells has been observed in previous studies using scRNA-seq analysis^11,25,26^. In some cases, the cells actively proliferated to enlarge the graft massively after implantation^8,13^. To reduce the number of non-endocrine cells, there are two approaches: concentrating only endocrine cells using cell sorting; and reducing non-endocrine cells through compound treatments or metabolic modification. The former approach, which assesses cells one by one, is useful for handling cells at a small scale^39^. Regarding the latter approach, chemical treatment, such as a YAP inhibitor, reduces the number of pancreatic progenitors via increased efficiency of endocrine cell induction^15^. This approach targets multiple cells concomitantly, in principle, and may be applicable for handling cells on a large scale. In the present study, we demonstrated that non-endocrine cell populations differentially express some genes, such as *FGFR2*, *PLK1/4* and *LDHB* (Fig. 4b), and that targeting the function of these genes with chemicals selectively reduced the number of non-endocrine cells (Fig. 4d,g). Because each of these methods is based on a different mechanism of action, combining previously reported methods with the findings obtained in this study may improve the cell quality in a large-scale culture.

We found that PD-166866 selectively reduced the number of non-endocrine cells mainly via the inhibition of FGFR2 in non-endocrine cells (Fig. 4a–e and Supplementary Fig. 8a). PD-166866 has been reported to increase *NGN3* expression via FGFR1 inhibition when added at the time of induction of pancreatic progenitor cells into endocrine cells^35^. In this study, the timing of PD-166866 treatment was different from that in the previous report, and PD-166866 was added after 4 days of induction of endocrine cells with a γ-secretase inhibitor (Supplementary Fig. 1). PD-166866 treatment during this period specifically decreased the number of non-endocrine cells without increasing the number of CHGA^+^ endocrine cells (Fig. 4d). Therefore, the effects of PD-166866 seen in the present study are probably different from the previous one and mediated directly by FGFR2 inhibition in non-endocrine cells.

In the PLK family, PLK1 is a well-studied molecule that is known to control the progression of the M phase in the cell cycle process^40^. In this study, *PLK1* was highly expressed in Cluster 10, in which the cell cycle was ongoing (Fig. 4b and Supplementary Fig. 5b), and treatment with a selective PLK1 inhibitor (GSK 461364) reduced the number of cells in Cluster 10, as expected (Fig. 4d). Unexpectedly, we found that the PLK1 inhibitor also reduced the number of non-endocrine cells in Clusters 5 and 13, in which *PLK1* was hardly expressed (Fig. 4b,d). We hypothesize that the non-biased scRNA-seq analysis classified cells in which the proliferation process was ongoing into Cluster 10. However, since the cells in Clusters 5 and 13 were more potent proliferation than endocrine cells, the cells entered the proliferation process during the 7-day treatment with the PLK1 inhibitor, resulting in the cells becoming targets of the PLK1 inhibitor. Thus, the PLK isoform inhibition is effective for removing proliferating and highly proliferative cells, in other words, is effective for selecting low-proliferative cell types.

Metabolic selection is an approach that removes off-target cells based on the difference in energy sources among cell types. Recent reports have demonstrated that metabolic selection reduces off-target cells, mainly composed of incompletely differentiated cells, in directed differentiation from pluripotent stem cells, such as cardiomyocytes, neural progenitors and even in definitive endoderm^41–43^. In this study, we demonstrated that the differential expression of *LDHB* in non-endocrine cells led to sufficient function because the number of non-endocrine cells was preferentially reduced by treatment with 2-DG (Fig. 4b,f,g). As inhibition of glycolysis is one of the approaches to remove proliferative cancer cells^44^, glycolysis inhibition has the potential to be an approach to remove unintended cells in directed differentiation towards less proliferative terminally differentiated cells.

We demonstrated three different approaches for non-endocrine cell-targeted reduction, namely inhibition of FGFR2, PLK1/4, and glycolysis. Through inhibition of these factors, five compounds (PD-166866, GSK 461364, CFI-400945, TR06141363, and 2-DG) showed specific reduction of non-endocrine cells (Fig. 4c–h). PD-166866 and TR06141363 were used to induce current iPIC; implantation results of iPICs suggested that these two compounds eliminated most potential non-endocrine cells while maintaining adequate in vivo efficacy (Fig. 1d–k and Supplementary Fig. 7). Therefore, PD-166866 and TR06141363 treatment have clinical application potentials. However, scRNA-seq data revealed that non-endocrine cells remained in current iPIC (Clusters 10 and 16), although very few (Supplementary Fig. 3b). This slight difference could have a significant impact in clinical applications, which require > 10^8^ order cells. When removal of non-endocrine cells is insufficient in iPIC in an over 10^8^ cell scale, further removal of these off-target cells might be achieved by adapting PLK or glycolysis inhibition strategy. The PLK inhibition strategy may have little impact on in vivo efficacy since GSK 461364 and CFI-400945 had little effect on endocrine cell number (Fig. 4d). This is also supported by the fact that iPIC differentiated following TR06141363 treatment, which has PLK4 inhibitory activity, showed sufficient in vivo efficacy (Fig. 1g–j and Supplementary Fig. 8b). Therefore, the PLK inhibition strategy is the first choice as additional removal of non-endocrine cells. In particular, GSK 461364 is promising due to its PLK1 inhibitory activity, which was not covered by TR06141363 (Supplementary Fig. 8b). For the glycolysis inhibition strategy, there is a limitation in using 2-DG, as observed in the current study: 2-DG treatment reduced not only non-endocrine cells but also endocrine cells, although the reduction rate of endocrine cells was smaller than that of non-endocrine cells (Fig. 4g). This is likely since 2-DG dose not target LDHB, which was specifically expressed in non-endocrine cells (Fig. 4b). We anticipate that LDHB subunit-specific inhibitors reduce the number of non-endocrine cells without affecting endocrine cells, although further studies are warranted.

In summary, we highlighted non-endocrine cells that are potentially contaminating in vitro-generated cells by directed differentiation from pluripotent stem cells. We showed that these non-endocrine cells consist of (1) heterogeneous proliferating cells, and (2) cells with not only pancreatic traits but also liver or intestinal traits, which are marked by *FGB* and *AGR2*. In addition, we demonstrated novel approaches for non-endocrine cell-targeted reduction, such as inhibiting FGFR2, PLK1/4, and glycolysis, which were predominantly activated in non-endocrine cells. Among the non-endocrine cell reduction candidates, PD-166866 and TR06141363 were used to induce current iPIC, and these compounds probably eliminated most of the potential non-endocrine cells while maintaining adequate in vivo efficacy. Although the iPIC safety assessment was inconclusive due to an insufficient in vivo sample size, our findings could contribute to mitigating the safety risks of iPIC for future clinical applications that require cell manufacturing on a large scale.

## Methods

### Cell culture and iPSC-derived pancreatic islet cells (iPIC) differentiation

Ff-I14s04 and QHJI-14s04 were kindly provided by the Center for iPS Cell Research and Application (CiRA), Kyoto University. Ff-I14s04 is derived from the same clone as QHJI but cultured and stocked for non-clinical use. QHJI-14s04 is a stock for clinical use. Cells were maintained on iMatrix-511 (Nippi)-coated dishes in StemFit AK03N (Ajinomoto) at 37 °C in a humidified 5% CO_2_ incubator. Cells were passaged every 3 or 4 days by non-enzymatic dissociation using 0.5 mM EDTA (Thermo Fisher Scientific) and subjected to differentiation experiments, usually after over 2 weeks of running culture. The use of human iPSCs was approved by the ethical review committee of Kyoto University and Takeda Pharmaceutical Company Limited. For differentiation culture to generate iPIC, we performed 2D monolayer to static aggregate culture based on our previous report^10^ and 3D stirred-floating aggregate culture. The details for 3D floating culture are provided below.

#### Stage 1

Dissociated undifferentiated iPSCs were resuspended at a density of 6 × 10^6^ cells in a spinner type 30 mL bio-reactor (Biott) suspended in AK03N containing 10 μM Y-27632 (FUJIFILM Wako) and stirred at a speed of 70 rpm throughout culture. The next day, aggregated cells were cultured in DMEM (high glucose, GlutaMAX Supplement, pyruvate; Thermo Fisher Scientific) or RPMI 1640 Medium (Thermo Fisher Scientific) supplemented with 1% (v/v) penicillin/streptomycin (P/S, FUJIFILM Wako), 1 × B-27 (Thermo Fisher Scientific), 1% Pluronic® F-68 (Poloxamer 188, Merck Millipore) to reduce fluid mechanical damage, 5–10 ng/ml^45^ Activin A (PeproTech), 3 μM CHIR99021 (Axon Medchem), and 1% DMSO (FUJIFILM Wako). The following day, CHIR99021 was removed from the medium, and culture was continued for another 2 days.

#### Stage 2

Cells were cultured with MCDB 131 medium (Thermo Fisher Scientific) supplemented with 1% P/S, 0.5 × B27, 1% Pluronic® F-68 and 50 ng/ml keratinocyte growth factor (KGF, R&D Systems), 4.44 mM glucose (added to yield a final concentration of 10 mM, FUJIFILM Wako), 1.5 g/L NaHCO_3_ (FUJIFILM Wako), and 1% GlutaMAX (Thermo Fisher Scientific) for 4 days.

#### Stage 3

Continuing culture was performed with improved MEM (iMEM, Thermo Fisher Scientific) containing 1% P/S, 0.5 × B27, 1% Pluronic® F-68, 50 ng/ml KGF, 100 ng/ml Noggin (FUJIFILM Wako), 0.5 μM 3-keto-N-aminoethyl-N'-aminocaproyldihydrocinnamoyl cyclopamine (KAAD-cyclopamine, Toronto Research Chemicals), and 10 nM 4-[(E)-2-(5,6,7,8-tetrahydro- 5,5,8,8-tetramethyl-2-naphthalenyl)-1-propenyl] benzoic acid (TTNPB, Santa Cruz Biotechnology) for 3 days.

#### Stage 4

Cells were exposed to iMEM containing 1% P/S, 0.5 × B27, 1% Pluronic® F-68, 100 ng/ml KGF, 50 ng/ml epidermal growth factor (EGF, R&D Systems), 10 mM nicotinamide (STEMCELL Technologies), 0.1 μM TR05991851 (Takeda original ROCK inhibitor), 0.5 μM phorbol 12,13-dibutyrate (PdBU, Merck Millipore), and 5 ng/mL activin A for 4 days.

#### Stage 5

Cells were treated with iMEM with 1% P/S, 0.5 × B27, 1% Pluronic® F-68, 0.25 μM SANT-1 (Merck), 50 nM retinoic acid (Merck), 10 μM ALK5 inhibitor II (Santa Cruz Biotechnology), 100 nM LDN-193189 (MedChemExpress), 1 μM L-3,3ʹ,5-triiodothyronine (T3, Merck Millipore), 50 ng/ml basic fibroblast growth factor (bFGF, PeproTech), 1 μM XAV939 (Merck Millipore), and 10 μM Y-27632 for 2 days.

#### Stage 6

Cells were cultured with iMEM containing 1% P/S, 0.5 × B27, 1% Pluronic® F-68, 1 μM γ-secretase inhibitor, RO4929097 (“GSI” in Supplementary Fig. 1, Chem Scene), 10 μM ALK5 inhibitor II, 100 nM LDN-193189, and 1 μM T3 for 4 days. To generate s6-iPIC-B, cells were treated with the same medium for another 7 days. In the case of s6-iPIC-A and iPIC, 1 μM PD-166866 (Merck Millipore) was added starting on the fourth day. In Fig. 4c–h, 3 μM GSK 461364 (Cayman), CFI-400945 (Apexbio), 1 μM TR06141363 (Takeda original multi-kinase inhibitor), and 2-deoxy-D-glucose (2-DG) were added as alternatives to PD-166866 in the s6-iPIC-A protocol. To generate iPIC, cells were dissociated on the seventh day at stage 6, seeded into Elplasia multi-well plates (4440, Corning) and cultured for the remaining 4 days in Stage 7 medium.

#### Stage 7

Stage 7 medium is based on Rezania et al. with some modifications. Cells were exposed to MCDB 131 medium with 1% P/S, 2% fat-free BSA (FUJIFILM Wako), 14.44 mM glucose (to generate a final concentration of 20 mM), 1.5 g/L NaHCO_3_, 1% GlutaMAX, 0.5% ITS-X (Thermo Fisher Scientific), 10 μM ALK5 inhibitor II, 1 μM T3, 10 μM ZnSO_4_ (Merck Millipore), 1.4 IU/ml heparin sodium salt (Nacalai Tesque), 1 mM N-acetyl cysteine (Merck Millipore), 10 μM Trolox (FUJIFILM Wako), 2 μM R428 (Selleck), 1 μM PD-166866, 3 μM TR06141363, and 10 μM Y-27632, for 4 days.

### Flow cytometry

Differentiation efficacy and quality at individual stages based on the developmental markers were analyzed with immunostaining methods and LSRFortessa X20 flow cytometry equipment (BD), as described previously^10^. Data were processed with FlowJo software. The primary antibodies are listed in Supplementary Table 1. Secondary antibodies of the appropriate species were conjugated to AlexaFluor 488, 546, 568 and 647 of appropriate species (Thermo Fisher Scientific or Jackson).

### Type 1 diabetes mouse model

NOD.CB17-Prkdc-scid/J (NOD-scid) mice were obtained from Charles River. Male mice between the ages of 8 and 9 weeks were intraperitoneally injected with multiple low doses of streptozotocin (STZ, 50 mg/kg/day for 5 days, Sigma). Mice that became hyperglycemic within 2–3 weeks after STZ injection were subjected to implantation experiments as a type 1 diabetes mouse model (STZ-NOD-scid mice). All animal studies were conducted at Shonan iPark, one of the AAALAC international accreditation facilities, and approved by the iPark institutional animal care and use committee. All experiments were performed in accordance with the relevant guidelines and regulations, including the ARRIVE guidelines.

### Implantation and in vivo assessment

Differentiated iPIC aggregates were mixed with 100 μL of fibrinogen/50 μL of thrombin solution, incubated at 37 °C for 5 min and then implanted in the subcutaneous space of anaesthetized STZ-NOD-scid mice (3–4 × 10^6^ cells/mouse). Fibrinogen from human plasma (Merck Millipore) and thrombin (Sigma) were reconstituted in iMEM and in PBS to make 10 mg/mL and 50 IU/mL solutions, respectively, and stored at − 80 °C until use. For the kidney capsule implantation study (Supplementary Fig. 7), s6-iPIC-A (1.4 × 10^6^ cells/mouse) or s6-iPIC-B (3.6 × 10^6^ cells/mouse) was implanted directly into the kidney capsule without fibrin gel. We monitored the blood glucose levels of implanted animals using an Accu-Chek Aviva system (Roche DC Japan) and collected plasma samples from the tail vein on the indicated days. For the oral glucose tolerance test, the mice were fasted overnight and orally injected with a 2 g/kg glucose solution (Otsuka), and plasma samples were collected from the tail vein before and 15, 30, 60 and 120 min after injection.

### Plasma glucose and hormone measurements

Plasma glucose, human C-peptide and mouse C-peptide levels were measured using the glucose test C-II Wako (Fujifilm Wako), Mercodia Ultrasensitive C-peptide ELISA (Mercodia) and mouse C-peptide measurement kit (Morinaga) according to the manufacturer’s instructions.

### Tissue processing and immunostaining

Implanted grafts were collected and fixed with 4% paraformaldehyde (FUJIFILM Wako) for over 24 h at 4 °C and embedded in paraffin or frozen in OCT compound. Paraffin blocks were sectioned at 5 μm and used for hematoxylin and eosin staining, Masson trichrome staining and immunostaining. Frozen blocks were sectioned at 10 μm and used for immunofluorescence staining. The primary antibodies are listed in Supplementary Table 1. Secondary antibodies were conjugated to AlexaFluor 488, 546 or 568 (Thermo Fisher Scientific or Jackson). Frozen sections were also counterstained with Hoechst (Thermo Fisher Scientific) to label the nucleus.

### Single-cell RNA sequencing library preparation, sequencing and data processing

A total of 6 samples (1 sample of iPIC, 3 samples of s6-iPIC-A, 1 sample of s6-iPIC-B, and 1 sample of reference human islet) underwent scRNA-seq. Human islets were purchased from PLODO. All 5 samples of three versions of iPICs were prepared from Ff-I14s04 line according to the inducers of Supplementary Fig. 1. iPICs were cultured from stage 1 with 3D stirred-floating culture with shear stress using bio-reactor (Biott). As an exception, only 1 of 3 samples of s6-iPIC-A was cultured in 2D monolayer from stages 1 to 4, and then in static aggregate culture without shear stress using non-adhesive V bottom 96 well plate (SUMITOMO BAKELITE) in accordance with our methods^10^. Single-cell RNA-seq libraries were generated using the 10 × Genomics Chromium™ controller and Chromium Single Cell 3' kits v2 (10 × Genomics) according to the manufacturer’s instructions. Successful cDNA amplification and library construction were ensured with High Sensitivity DNA kits on an Agilent 2100 Bioanalyzer (Agilent). The obtained libraries were sequenced using Hi-seq (Illumina) with 150 bp paired-end reads at a depth of > 100,000 reads per cell. Sequencing reads were aligned to the human GRCh38 genome reference, and gene counts were quantified as UMIs using Cell Ranger v2.0.1 (10 × Genomics). We imported UMI count matrices into the R v3.3.1 software Seurat v2.0.1 package^46,47^, where normalization was performed according to the package’s default setting. Cells with mitochondrial gene counts over 10% were regarded as dead or damaged cells and removed for further analyses. UMI count matrices were scaled by regressing out the number of total UMI counts per cell and the percentage of mitochondrial gene counts. Genes for dimensional reduction were selected by the average expression and dispersion of each gene, and principal component analysis was performed. Principal components were used for Seurat’s shared nearest neighbor graph clustering and t-distributed stochastic neighbor embedding (*t*-SNE) dimensional reduction to create a visualization of data. The cell cycle was evaluated and scored using the expression of genes known as S-phase, G1, and G2M markers. To estimate cell types and similarities within iPICs with reference to human organs, we performed reference component analysis (RCA) using the RCA v1.0.0 package^31^. Differential gene expression analysis of each cluster compared with the others was performed using the likelihood-ratio test for single-cell gene expression in Seurat. For trajectory analysis, processed UMI count matrices were imported to a single-cell dataset for the Monocle v2.6.3 package^28–30^. We selected the genes for ordering cells with ‘dpFeature’ in monocle and contracted the single-cell trajectories via the ‘DDRTree’ algorithm.

### Measurement of inhibitory effects on FGFR and PLK isoforms

The percent (%) inhibition of FGFR and PLK isoforms by PD-166866, TR06141363, GSK 461364, and CFI-400945 was evaluated with a time-resolved fluorescence resonance energy transfer (TR-FRET)-based competitive binding assay^36^, performed using 1536-well, white, flat-bottomed plates (Greiner Bio-One). Assay buffer comprised 50 mmol/L HEPES, 10 mmol/L MgCl2, 1 mmol/L EGTA, 0.1 mmol/L DTT, and 0.01% Brij-35. Test compounds (1 and 0.1 µmol/L) were mixed with recombinant kinase proteins with an epitope tag, a terbium-labelled anti GST-tag antibody, and fluorescent labelled ligands. After 1 h incubation at room temperature, TR-FRET signals were measured with EnVision (PerkinElmer). DMSO and 5 µmol/L control inhibitor (Staurosporine or other multi kinase inhibitors) were used as 0 and 100% controls, respectively. The percent (%) inhibition was calculated based on the signals of the 0% and 100% inhibition samples in the absence and presence of control inhibitors, respectively.

### Analysis of mRNA expression by quantitative real-time PCR

After iPIC differentiation, cDNA samples were synthesized from lysates using TaqMan Gene Expression Cells-to-Ct kits (Thermo Fisher Scientific) according to the manufacturer's instructions, followed by quantitative real-time polymerase chain reaction analysis using a Prism 7900HT sequence detector (Thermo Fisher Scientific). The thermal cycling parameters were 2 min at 50 °C and 10 min at 95 °C, followed by 40 cycles at 95 °C for 15 s and 60 °C for 1 min. The mRNA levels were analyzed with the comparative Ct method (2-ΔΔCt) using *RPLP0* as the housekeeping gene. The TaqMan Gene Expression assays (Thermo Fisher Scientific) used herein were as follows: Hs00170586_m1 (*FGB*), Hs00356521_m1 (*AGR2*), and Hs00420895_gH (*RPLP0*).

### Statistical analysis

Dunnett's multiple-comparison test was performed at a significance level of *P* < 0.05 to determine statistical significance in Figs. 3c,d, 4c,e. Additionally, the Aspin-Welch test or Student's t-test was performed at a significance level of *P* < 0.05 to determine statistical significance between two groups in Fig. 3c,d. The dose–response relationships in Fig. 4f,h were tested using Williams or Shirley-Williams tests with a one-tailed significance level of *P* < 0.025 based on the results of the homogeneity of variance test (Bartlett’s test). All statistical analyses were performed using Statistical Analysis System version 9.3 (SAS Institute, NC, USA).

## Supplementary Information

Supplementary Information.

## Data Availability

The data that support the findings of this study are available from the corresponding author upon reasonable request. Single-cell RNA sequencing data will be deposited in the Gene Expression Omnibus (GEO).
