# Supplementary material for: Characterization and reduction of non-endocrine cells accompanying islet-like endocrine cells differentiated from human iPSC
Source: Sci Rep. 2022 Mar 18;12:4740. doi: 10.1038/s41598-022-08753-5 (PMC8933508; doi:10.1038/s41598-022-08753-5)
Supplement: Supplementary file 1 — Supplementary Information. [file 41598_2022_8753_MOESM1_ESM.docx]

**Supplementary information**

**Characterization and reduction of non-endocrine cells accompanying islet-like endocrine cells differentiated from human iPSC**

Hideyuki Hiyoshi^1,3,#,*^, Kensuke Sakuma^1,3,4,#^, Noriko Tsubooka-Yamazoe^1,3,4,#^, Shinya Asano^5^,

Taisuke Mochida^1,3^, Junji Yamaura^3,6^, Shuhei Konagaya^2,3,4^, Ryo Fujii^5^, Hirokazu Matsumoto^1,3^,

Ryo Ito^1,3,4^ and Taro Toyoda^2,3,*^

^1^T-CiRA Discovery, Research, Takeda Pharmaceutical Company Limited, Fujisawa, Kanagawa, Japan.

^2^Department of Cell Growth and Differentiation, Center for iPS Cell Research and Application (CiRA), Kyoto University, Kyoto, Japan.

^3^Takeda-CiRA Joint Program for iPS Cell Applications (T-CiRA), Fujisawa, Kanagawa, Japan.

^4^Orizuru Therapeutics, Inc., Fujisawa, Kanagawa, Japan

^5^Axcelead Drug Discovery Partners, Inc., Fujisawa, Kanagawa, Japan.

^6^Pharmaceutical Science, Takeda Pharmaceutical Company Limited, Fujisawa, Kanagawa, Japan.

^#^These authors contributed equally to this work.

*Co-corresponding authors

**
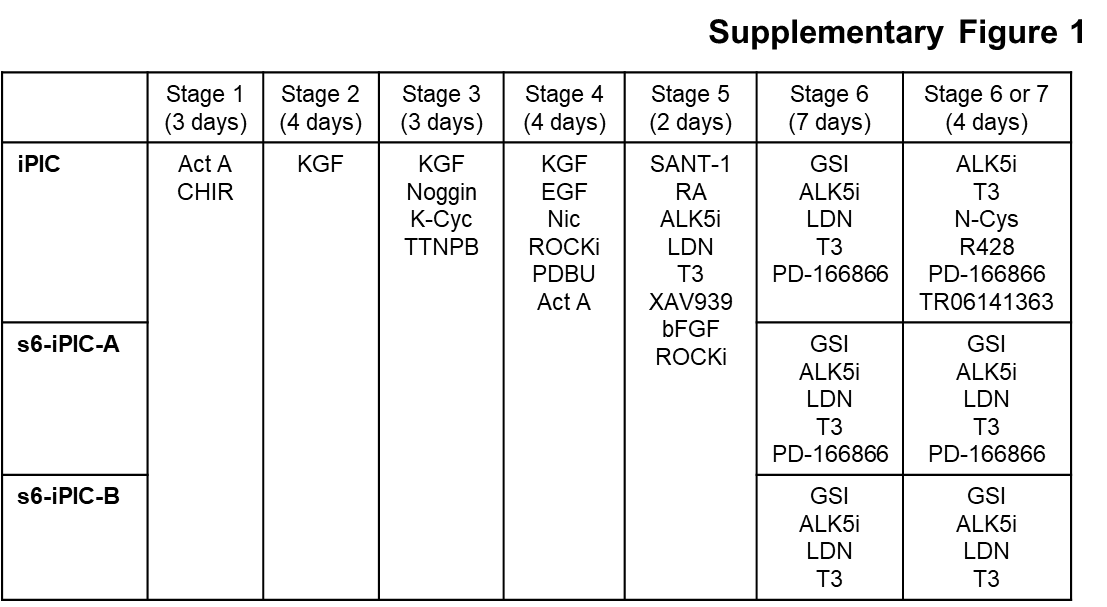
**

**Supplementary Figure 1 | A Schematic diagram of the differentiation reagents for three types of iPICs.**

Act A; activin A, CHIR; CHIR99021, K-Cyc; KAAD-cyclopamine, Nic; nicotinamide, ROCKi; ROCK inhibitor, RA; retinoic acid, ALK5i; ALK5 inhibitor II, LDN; LDN-193189, GSI; γ-secretase inhibitor, N-Cys; *N*-acetyl cysteine.


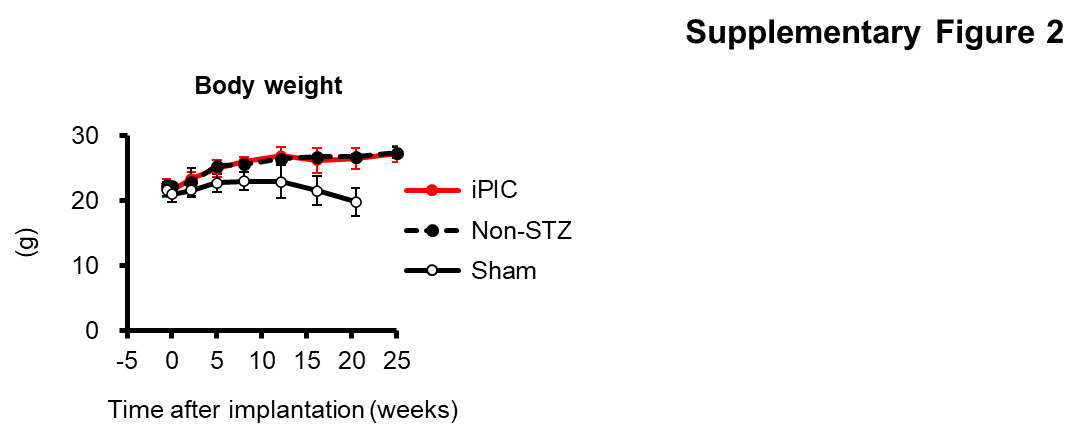


**Supplementary Figure 2 |** **Body weight change during implantation study in Figure 1.**

Body weight change following iPIC (3 × 10^6^ cells/mouse) implantation. Data are shown as the mean ± SD (iPIC; n = 5→3, Non-STZ; n = 4→3, sham; n = 4→3). The decrease in n number is due to unexpected death.


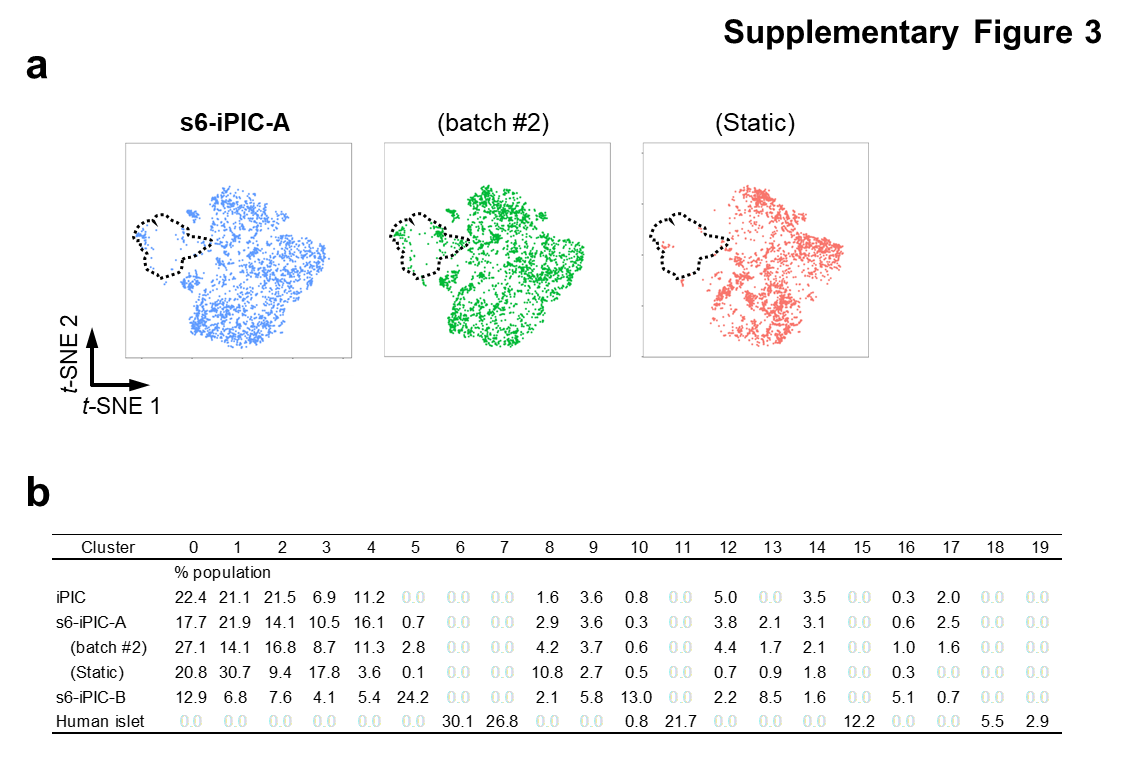


**Supplementary Figure 3 | Additional information on single-cell RNA sequencing samples.**

**a**, Individual *t*-SNE projections for the first batch of s6-iPIC-A (which is also shown in Fig. 2c as a representative sample) and two additional s6-iPIC-A samples (batch #2 and static culture). The dotted line in black represents the non-endocrine population from the iPICs.

**b**, Percentage of cells in the clusters classified in Fig. 2d. Of note, in iPIC, cells were not detected in Clusters 5 and 13, while cells were detected in proliferative cluster 10 across all iPICs and islet samples.


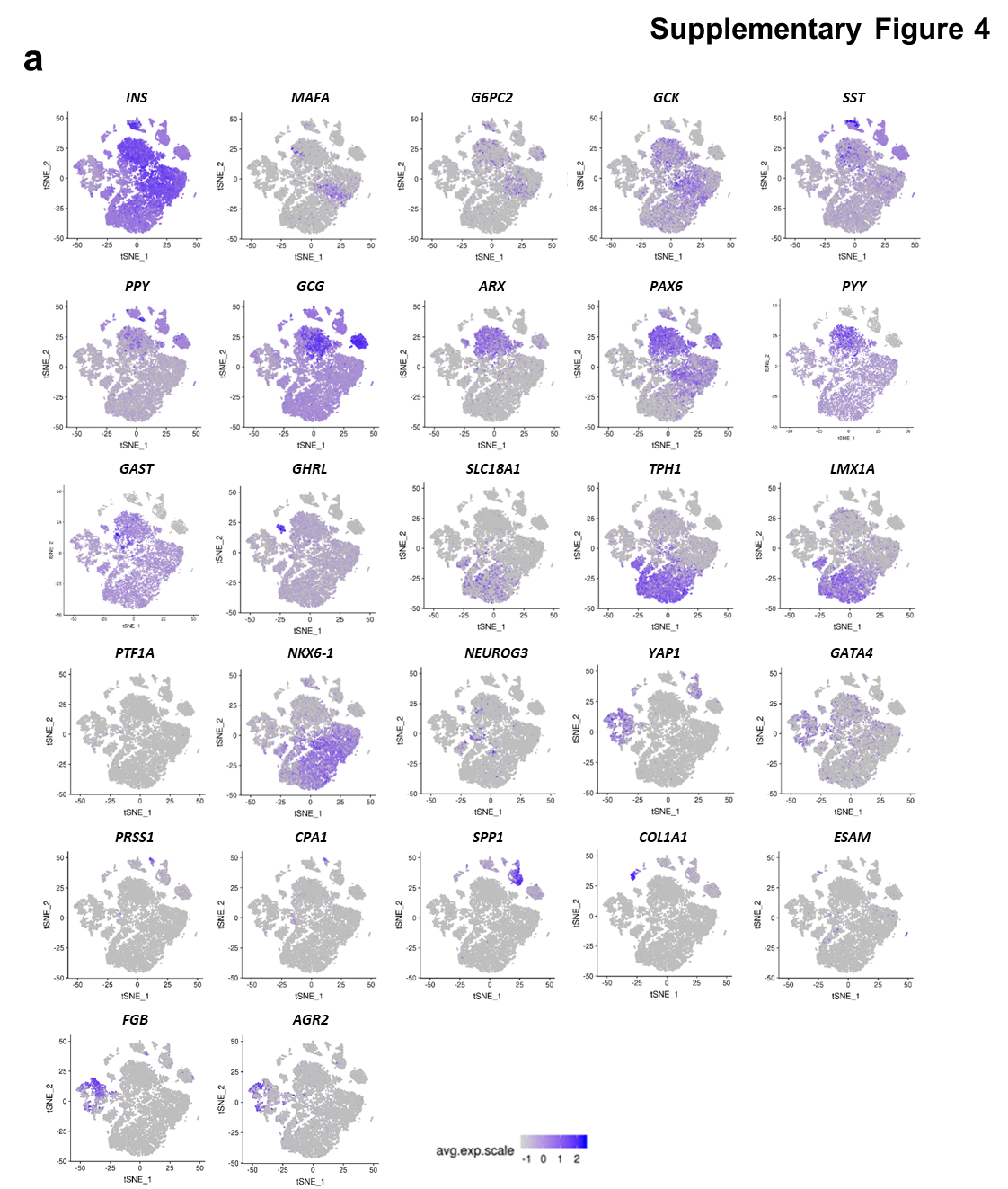


**
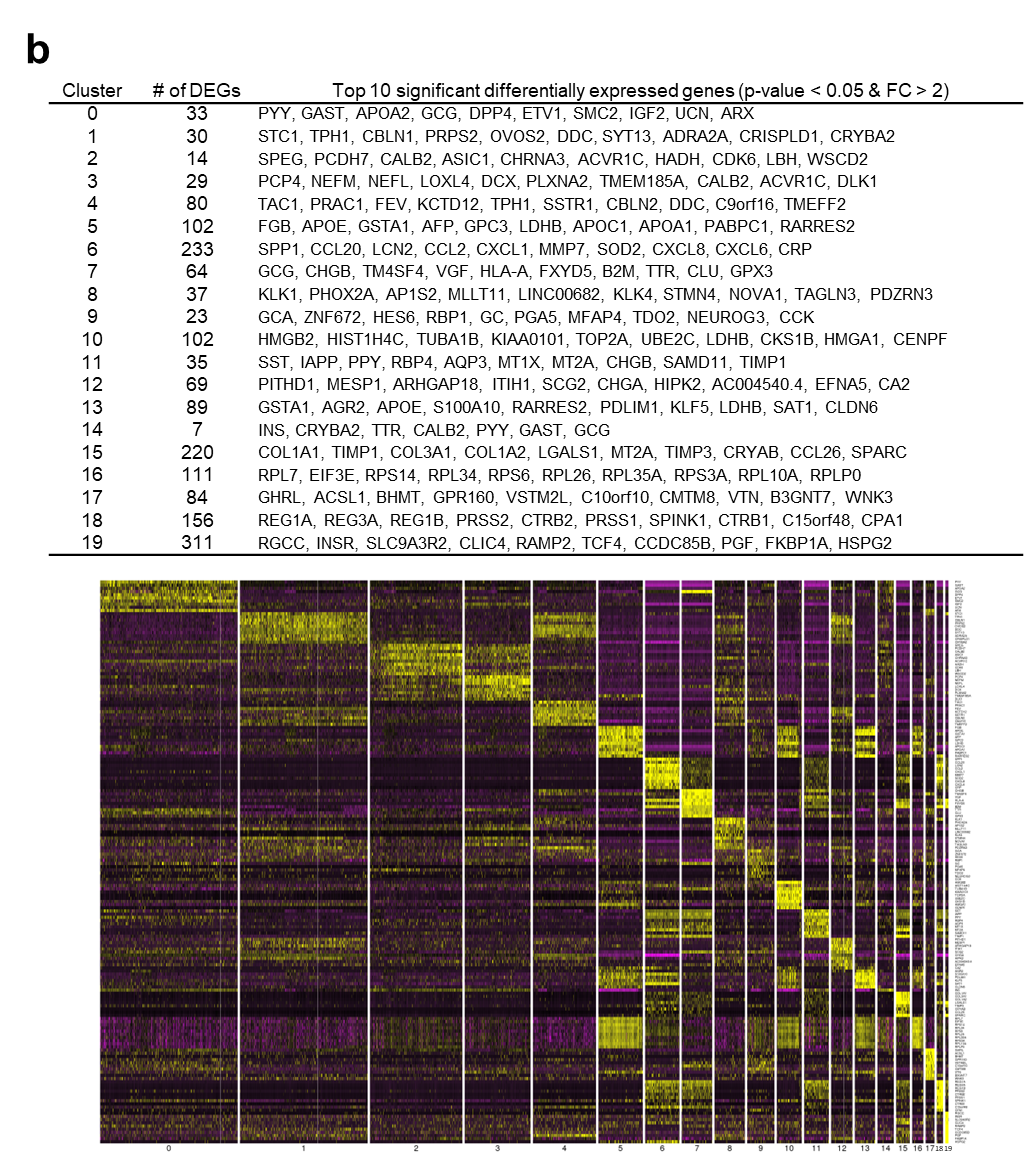
**

**Supplementary Figure 4 | Expression distribution of known cell type-specific markers on *t*-SNE projections and differentially expressed genes in each cluster.**

**a**, Single-cell expression of remarkable cluster determinant markers in iPICs and human islets.

**b**, Most significant differentially expressed genes (DEGs), with a greater than 2-fold change and statistical significance of *p* < 0.05 as determined by the likelihood ratio test for single-cell expression.


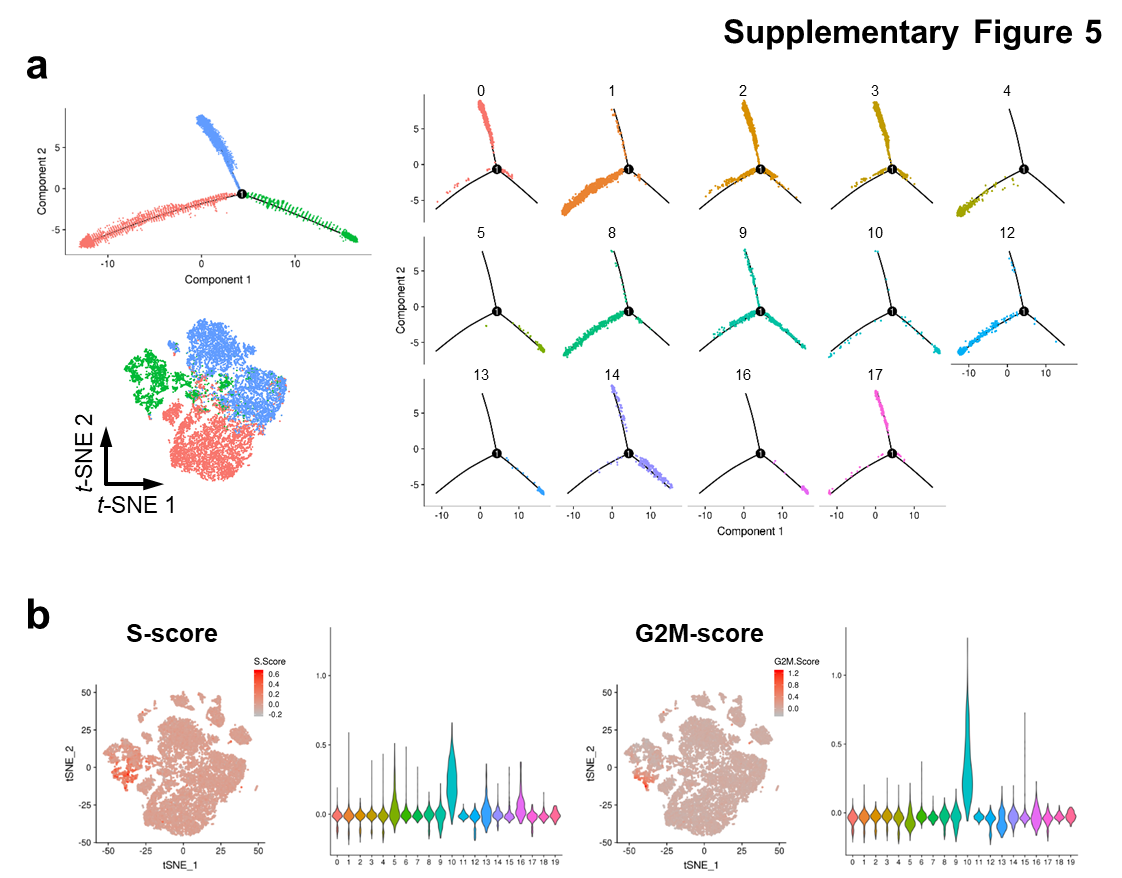


**Supplementary Figure 5 | Multifaceted analyses focusing on non-endocrine cells.**

**a,** Single-cell trajectory for cells composing iPICs reconstructed with *Monocle 2* to describe the progression of the differentiation process. The upper left panel shows the trajectory projection of the reconstructed iPICs with one branching point. The right panels show the position of cells composing each cluster classified in Fig. 2d on the trajectory projection. The lower left panel shows the trajectory analysis results reflected on the *t-*SNE projection.

**b,** Cell cycle phase assignments based on S-phase, G2 and M gene signatures indicated a highly proliferative population in red within the combined *t*-SNE plot and upward shifts in the violin plot.

**
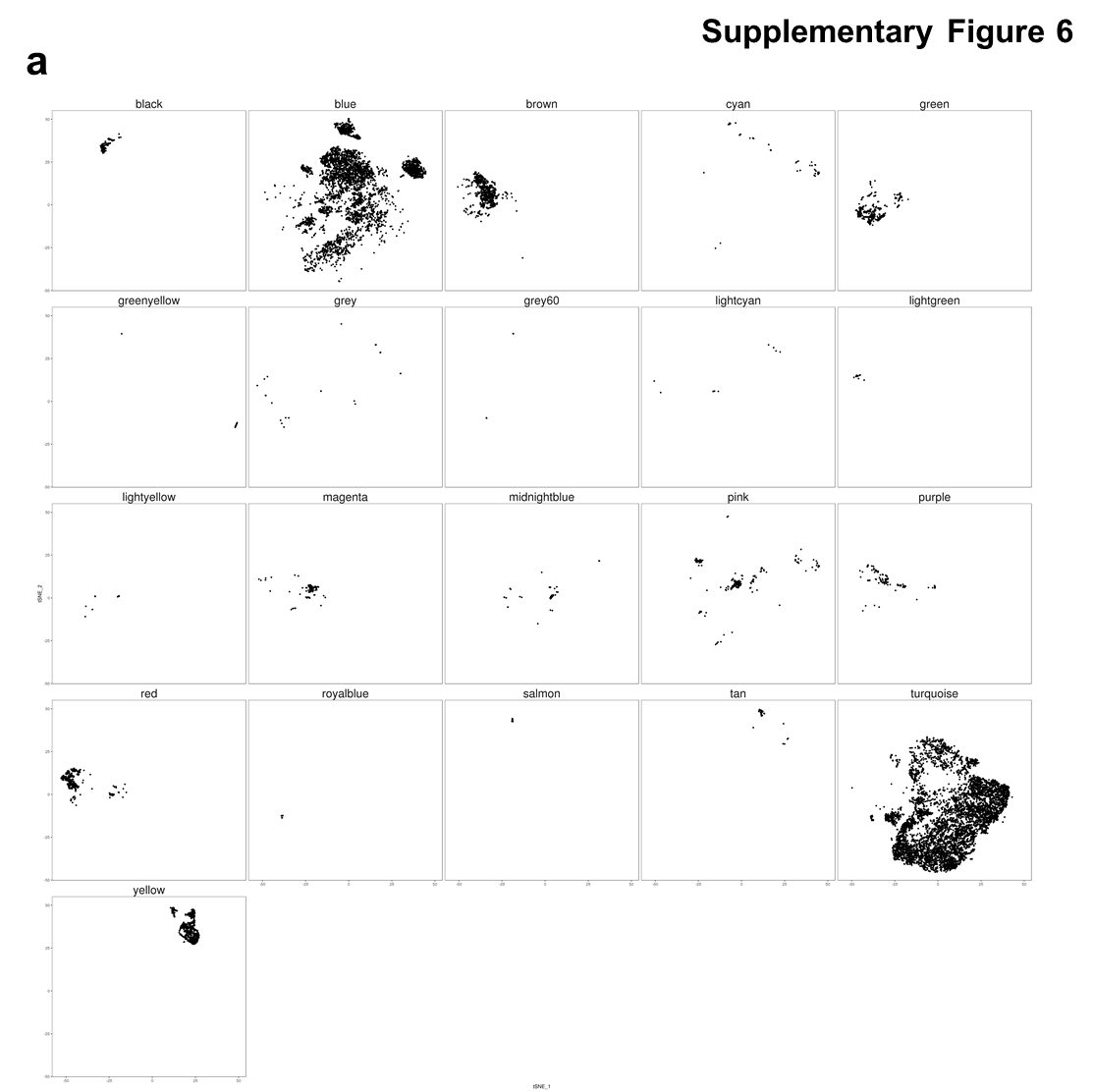
**

**
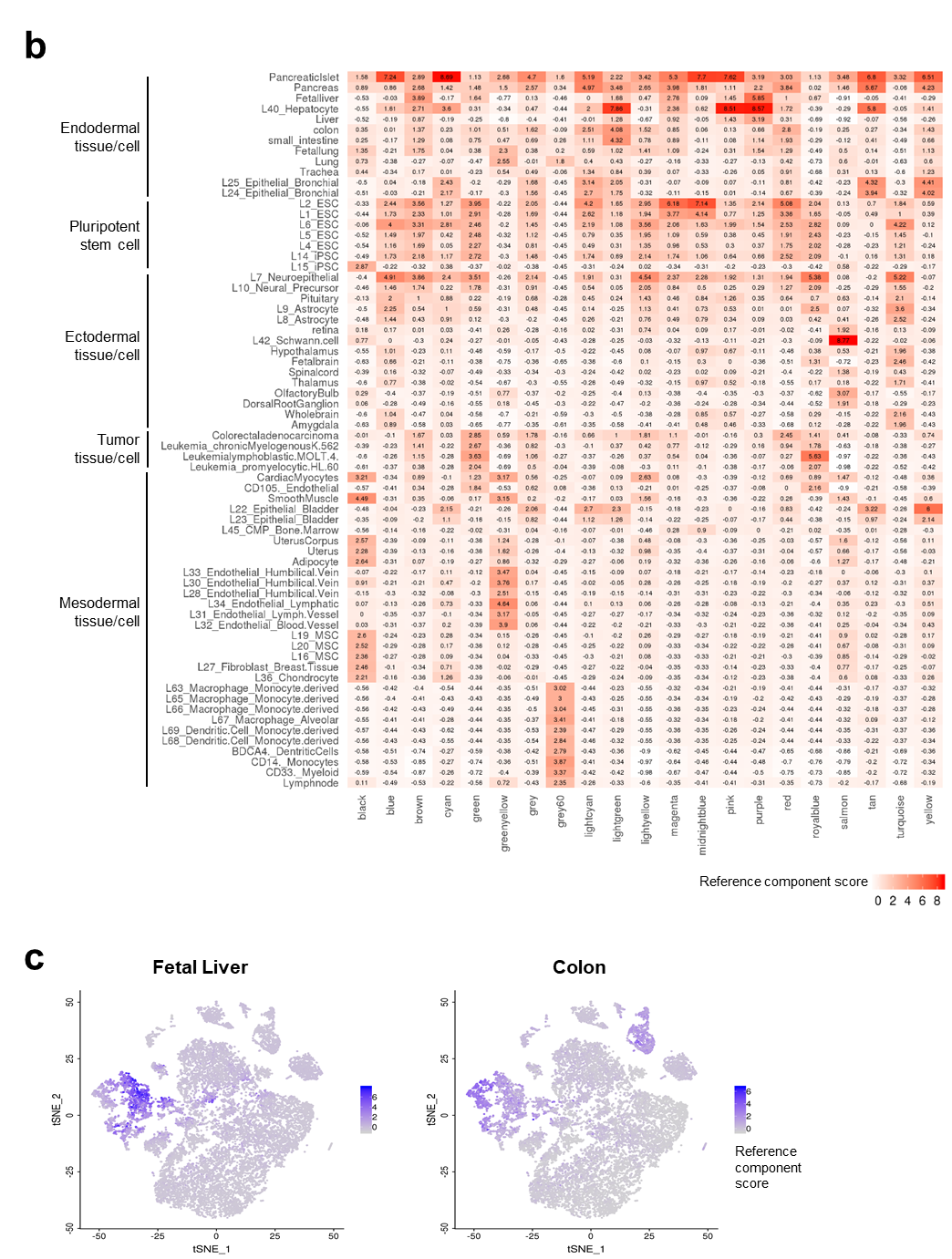
**

**Supplementary Figure 6 | Additional information for RCA.**

**a**, Distribution of each color in RCA on the *t-*SNE projection.

**b**, Heatmap of similar tissues or cell lines for each cluster classified by RCA.

**c**, Cellular distribution of reference component scores for fetal liver and colon references.


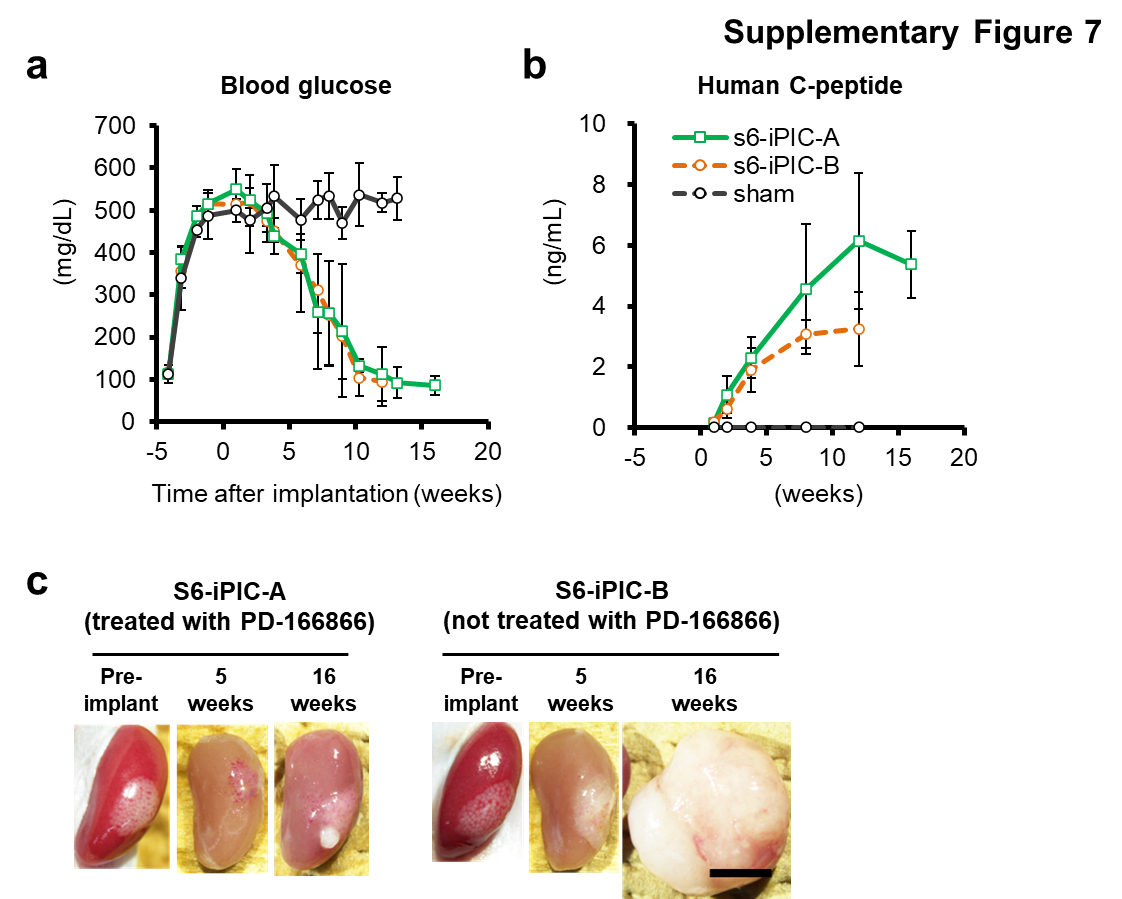


**Supplementary Figure 7 |** **Kidney capsular implantation of s6-iPIC-A and s6-iPIC-B.**

**a** and **b**, Blood glucose and plasma human C-peptide levels after s6-iPIC-A (1.4 × 10^6^ cells/mouse) or s6-iPIC-B (3.6 × 10^6^ cells/mouse) implantation. Data are shown as the mean ± SD (s6-iPIC-A; n = 4→3, s6-iPIC-B; n = 4→3, sham; n = 5→4). The decrease in n number is due to unexpected death.

**c**, Macroscopic changes of grafts post implantation; n = 1 at each time point. The graft of s6-iPIC-A showed slight hypertrophy at 16 weeks post implantation. In contrast, the graft of s6-iPIC-B gradually became larger than the kidney at 16 weeks post implantation. Reproducibility was confirmed in several similar experiments. Scale bar, 5 mm.


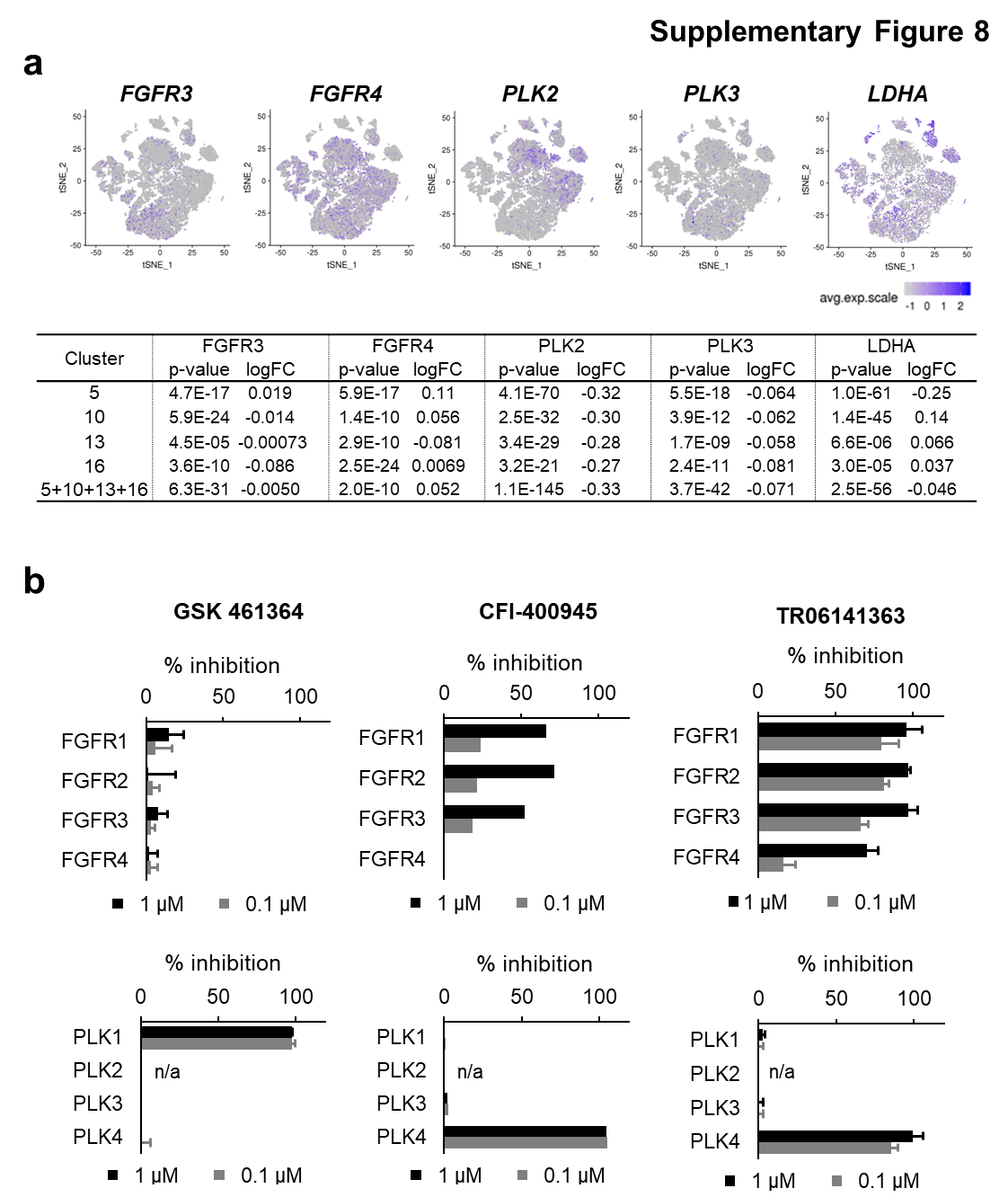


**Supplementary Figure 8 | Single-cell gene expression related to Figure 4b and inhibitory activities of GSK 461364, CFI-400945 and TR06141363 for FGFR and PLK isoforms.**

**a**, Single-cell gene expression of *FGFR3*, *FGFR4*, *PLK2*, *PLK3* and *LDHA*. The table contains the *p*-value and fold change in each indicated non-endocrine cluster compared to the others.

**b**, Inhibitory activities of GSK 461364, CFI-400945 and TR06141363 for FGFR and PLK isoforms in the TR-FRET-based competitive binding assay. Data are shown as the mean ± SD (n = 3-4, collected from independent experiments), the mean without SD bar (n = 2, collected from independent experiments) or raw value (n = 1). n/a; not available.

**Supplementary Table 1. List of primary antibodies used in immunofluorescence staining.**
